# Distribution of Bound Conformations in Conformational Ensembles for X-ray Ligands Predicted by the ANI-2X Machine Learning Potential

**DOI:** 10.1021/acs.jcim.3c01350

**Published:** 2023-10-30

**Authors:** Fengyang Han, Dongxiao Hao, Xibing He, Luxuan Wang, Taoyu Niu, Junmei Wang

**Affiliations:** † Department of Pharmaceutical Sciences and Computational Chemical Genomics Screening Center, School of Pharmacy, University of Pittsburgh, Pittsburgh, Pennsylvania 15261, United States; ‡ School of Electronics and Information Engineering, Ankang University, Ankang 725000, China

## Abstract

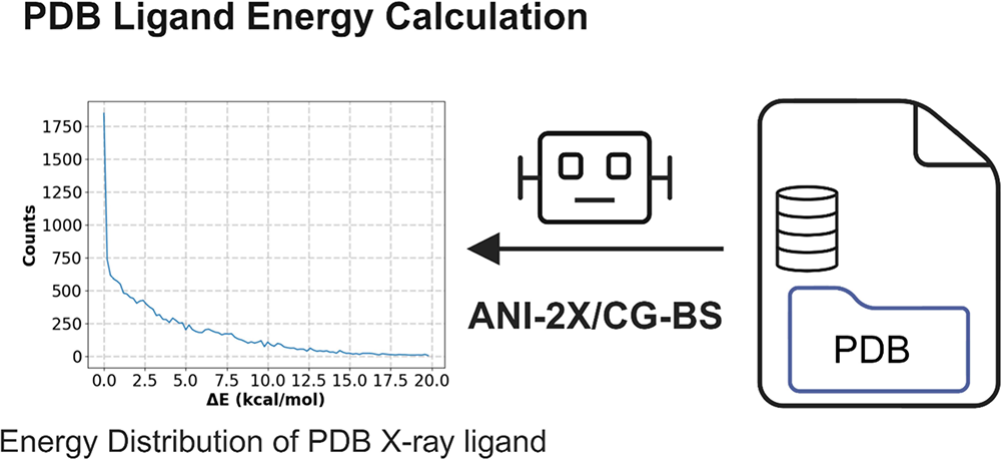

In this study, we systematically studied the energy distribution of bioactive conformations of small molecular ligands in their conformational ensembles using ANI-2X, a machine learning potential, in conjunction with one of our recently developed geometry optimization algorithms, known as a conjugate gradient with backtracking line search (CG-BS). We first evaluated the combination of these methods (ANI-2X/CG-BS) using two molecule sets. For the 231-molecule set, ab initio calculations were performed at both the ωB97X/6-31G(d) and B3LYP-D3BJ/DZVP levels for accuracy comparison, while for the 8,992-molecule set, ab initio calculations were carried out at the B3LYP-D3BJ/DZVP level. For each molecule in the two molecular sets, up to 10 conformations were generated, which diminish the influence of individual outliers on the performance evaluation. Encouraged by the performance of ANI-2x/CG-BS in these evaluations, we calculated the energy distributions using ANI-2x/CG-BS for more than 27,000 ligands in the protein data bank (PDB). Each ligand has at least one conformation bound to a biological molecule, and this ligand conformation is labeled as a bound conformation. Besides the bound conformations, up to 200 conformations were generated using OpenEye’s Omega2 software (https://docs.eyesopen.com/applications/ omega/) for each conformation. We performed a statistical analysis of how the bound conformation energies are distributed in the ensembles for 17,197 PDB ligands that have their bound conformation energies within the energy ranges of the Omega2-generated conformation ensembles. We found that half of the ligands have their relative conformation energy lower than 2.91 kcal/mol for the bound conformations in comparison with the global conformations, and about 90% of the bound conformations are within 10 kcal/mol above the global conformation energies. This information is useful to guide the construction of libraries for shape-based virtual screening and to improve the docking algorithm to efficiently sample bound conformations.

## 1 Introduction

The utilization of docking methodologies to explore numerous ligand conformations and their interactions with a receptor, thereby elucidating binding modes, represents a prevalent practice within virtual screening workflows.^1,2^ Upon conducting scoring and ranking analyses of the docking outcomes, researchers can acquire a collection of prospective hits for further analysis. In this workflow, the ultimate efficacy and desirability of the final hits are heavily influenced by two crucial upstream processes, namely, conformation generation and filtering.^3^ The generation of diverse conformations allows a more comprehensive examination of the conformational space accessible to a ligand, increasing the probability of obtaining the optimal protein–ligand binding pose. The conformation space of a molecule results from its rotatable bonds, which are a major source of molecular flexibility. Given the exponential expansion of the conformational space with an increase in the number of rotatable bonds, it is not wise to blindly increase the quantity of generated conformations to comprehensively explore the entire conformational space. Hence, guidance on how to generate a conformation ensemble for a molecule that contains the bound conformation is crucial in virtual screening and conformational library design.

Conformational energy serves as a robust evaluation metric that effectively captures a conformation’s relative stability. A molecule can adopt a conformation distinct from the global conformation when it interacts with a biomolecule to achieve a conformational energy of the complex lower than otherwise. However, the price of adopting a higher energy conformation must be compensated by the formation of a favorable complex conformation. Hence, it is imperative to establish a threshold to eliminate those conformations which are unlikely to be bound conformations in docking simulation and conformational library design.^4,5^ Perola et al. reported that changes and rearrangements to high-energy conformations up to 9 kcal/mol higher than the global minimum are sometimes allowed without significantly compromising binding affinity to the target.^6^ Their results were obtained from the two different force fields, OPLS-AA and MMFF. Their work also suggests that the optimal conformation may be overlooked when the energy threshold is set too low. However, their conclusion was reached using only 150 ligands.

The X-ray crystal structures obtained from the Protein Data Bank (PDB) database revealed many complex conformations with bound ligands. They serve as good references for unveiling the distribution of energy thresholds. For a PDB ligand, the conformation bound to a protein or nucleic acid target is considered a “bound conformation”. In the realm of conformational energy calculations, there are two main approaches based on either molecular mechanics (MM) or quantum mechanics. The former exhibits notable computational efficiency, yet its accuracy is constrained by the quality of the employed force-field model. On the other hand, the ab initio quantum mechanics method has shown high accuracy in describing the conformational energies but with a much high computational resource and time cost rendering them unpractical for large-scale library construction. In a previous study conducted by Sitzmann et al.,^7^ efforts were taken to describe the energy difference distribution of bound and global conformations based on density functional theory (DFT) methods at the level of B3LYP/6-31G(d). A total of 415 small molecules from the 2008 version of Ligand Expo were successfully calculated by Gaussian 03. This study demonstrated that the conformational energy changes of small molecules bound to proteins are within the range of 0 to approximately 25 kcal/mol. The computational resources and time expenses associated with conducting DFT calculations impose limitations on the scope of their research endeavors as the utilization of a small data set may potentially compromise its representativeness.

ANI (Accurate Neural network engIne for molecular energies) is an extensible neural network potential method that was proposed by Smith et al. at the University of Florida. This method achieves a good balance between the MM and the DFT methods in terms of speed and accuracy.^8^ However, upon combining the ANI-generated potential with Gaussian16 software, issues arise in performing geometry optimization and calculating the potential energy surface (PES).^9^ This is due to the insufficient smoothness of the PES generated by the machine learning model, which can readily lead to nonconvergence in geometry optimization. Such nonconvergence renders this combination inadequate for the computational estimation of conformational energies. The feasibility of utilizing the ANI methodology for large-scale computations has also been questioned due to the convergence issues, and without solving the problem, DFT approaches remain necessary for acquiring satisfactory distributions of torsional energies.^10^ In response to these challenges, we recently developed four novel geometry optimization algorithms in conjunction with ANI or other machine learning-based potentials. This development has enabled ANI to achieve overall satisfactory results in geometry optimization and PES scanning, in comparison with the DFT results. The conjugate gradient backtracking line search (CG-BS) algorithm has been identified as the most robust method in the optimization of 160 CHON molecules (atom types supported by ANI-1X) using both ωB97X/6-31G(d) and ANI-1X-CG-BS. The resulting analysis revealed an average root-mean-square deviation (RMSD) of only 0.05 Å. Moreover, an impressive 90.9% of conformational differences between the two methods fell below the threshold of 0.1 Å.

**Figure 1:**
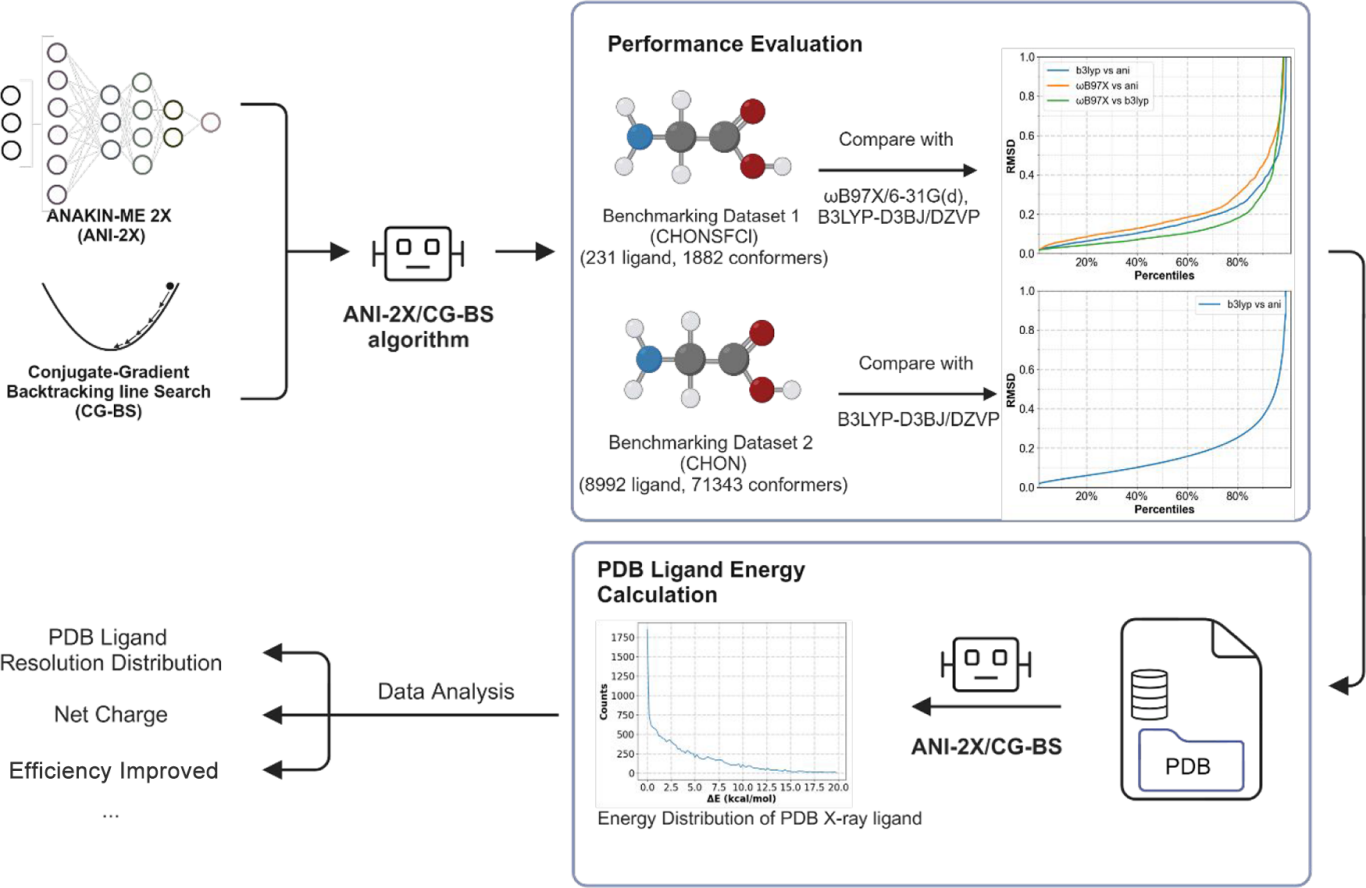
Overall workflow of studying the distribution of bound conformations in conformational ensembles for PDB ligands.

We will conduct evaluations of combination of the ANI-2X and CG-BS method (ANI-2x/CG-BS) for molecule energy calculation using two molecular data sets to attain our objective of obtaining guidance on the conformation library construction based on energy: a 231-molecule set for which geometry optimization was performed at both ωB97*X*/6-31G(d) and B3LYP-D3BJ/DZVP levels and an 8,992-molecule set for which geometry optimization was performed only at the B3LYP-D3BJ/DZVP level. Next, we will generate conformational ensembles for all of the selected PDB ligands using Omega2. We then conduct ANI-2x/CG-BS optimization for all of the Omega2-generated conformations as well as the bound conformation from the X-ray structures. Last, we will conduct conformational energy analyses and calculate the RMSD between the initial geometries from B3LYP-D3BJ/DZVP and optimized geometries from both ωB97X/6-31G(d) and ANI-2x/CG-BS. Based on the results of energy analysis, we will come up with a threshold for the effective generation of conformational ensembles containing bound conformations. The overall workflow for this work is summarized in Figure 1. We believe this information can provide guidance on both conformational library design and docking pose prediction algorithm development.

## 2 Methodologies

### 2.1 Molecule Data Sets for ANI-2X/CG-BS Evaluation

We assessed the effectiveness and accuracy of the ANI-2X/CG-BS method for calculating conformation energies and optimizing molecular geometries using 8992 small molecules from the Open Force Field Consortium (OpenFF) development team.^11,12^ Up to 10 conformations for each molecule were generated and optimized at the B3LYP-D3BJ/DZVP level using the Psi4 software package.^13^ The total number of conformations is 71,343 with an average of 7.93 conformations per molecule. These organic compounds of interest were contributed by 10 industrial companies; thus, this data set is anticipated to reflect the ongoing effort in small-molecule drug discovery. All molecules in this data set contain only C, H, N, O, F, S, and Cl elements which are covered by ANI-2x. Considering ANI-2x was developed against ωB97X/6-31G(d),^8^ we constructed a subset, which was designated as Benchmark Data set 1, that comprises 1882 conformations from 231 molecules. The 8992-molecule set was designated as Benchmark Data set 2. The Smiles string of every molecule in Benchmark Data set 2 is listed in Table S1. For each conformation in Benchmark Data set 1, we performed ωB97X/6-31G(d) optimization using the Gaussian 16 software package.^14^ We conducted ANI-2X/CG-BS optimization using an internal python program^9^ for all the bound and ensemble conformations. For the sake of convenience, we used the following abbreviations in the rest of the text: ωB97X for ωB97X/6-31G(d), B3LYP for B3LYP-D3BJ/DZVP, and ANI-2X for ANI-2X/CG-BS.

### 2.2 PDB Ligand Data Set

We collected ligands which have bound conformations from the PDB Ligand Expo, formerly known as Ligand Depot (http://ligand-expo.rcsb.org). This Web site provides chemical structure information pertaining to all small ligand molecules present in the PDB.^15^ The data set comprised a total of 36,104 ligand molecules distributed across 870,284 X-ray structure entries by December 2022. Additionally, 1,036 ligand molecules were present within 119,296 NMR structure entries, while 2060 ligand molecules were encompassed within 226,060 PDB entries obtained using other methodologies, such as electron microscopy.

We first selected ligand structures from X-ray structures for which their quality could be measured using resolutions. For every ligand molecule that was recorded in multiple complexes, only the one with the highest resolution was selected to extract its bound conformation for data analysis.

### 2.3 Conformation Generation

For each PDB ligand, we generated a conformational ensemble using OpenEye’s Omega2 module. This module adopts an energy-based approach to generate the possible conformations of a small molecule.^16^ These ligand molecule files were extracted from the PDB database in mol2 format with their chirality being reserved. Moreover, the applied conformational generation process did not alter the absolute configurations (*R*/*S* or *E*/*Z*) of a ligand molecule if it is a stereoisomer or geometric isomer. We applied the default setting and generated up to 200 conformations for each molecule. All of the conformations including the bound conformation from the PDB ligand database were subjected to ANI-2x optimization. By comparing the energies of the different conformations, we were able to identify the conformation with the smallest conformational energy, which was most likely the global minimum conformation in the conformational ensemble. These “global” conformations were employed to determine the relative energy values among the various conformations in each conformational ensemble for data analysis.

In the process of conformational generation, a certain alternation on a molecular structure may occur, such as the introduction of hydration atoms to fill in the open valence. Moreover, a change may occur on the atomic order and atom names. However, since the conformational generation process of the software leads to alterations in atomic sequence order as well as atom and bond information, it is difficult to identify and filter out abnormal molecules straightforward. The match_atomname program in the Antechamber software package was applied to judge if the Omega2-processed conformations and the bound conformations are the same molecule.^17^ A ligand was discarded if match_atomname found the bound conformation, and Omega2-processed conformations belonged to different molecules. Subsequently, the ligands lacking multiconformations were eliminated as the presence of only one conformation made them unsuitable for conformational energy distribution analysis. Owing to the capability of conformational software and the inherent limitations in the generation of conformations, it is important to acknowledge that the entirety of conformational space cannot be fully explored. To facilitate energy comparison, we use *E*_min_ and *E*_max_ to denote the minimum and maximum ANI-2X energies of the conformations in the conformational ensemble. We also use *E*_active_ to refer to the ANI-2X energy of the bound conformation. There are three possible scenarios: (i) *E*_active_ < *E_min_*; (ii) *E*_min_ ≤ *E*_active_ ≤ *E*_max_; and (iii) *E*_max_ < *E*_active_. We will mainly focus on the second scenario.

### 2.4 Ab Initio Calculations for the 231-Molecular Set

The conformational geometries of both data sets were previously optimized using the Psi4 program at the B3LYP-D3BJ/DZVP level. In this study, we carried out optimization of Benchmark Data set 1 at the ωB97X/6-31G(d) level with Gaussian16 software.^14^ When compared to the result obtained by B3LYP, this method takes dispersion forces into consideration, which is more accurate in the conformational energy calculation. These two ab initio results from DFT, ωB97X and B3LYP, served as the reference for performance evaluation in comparison to ANI-2x/CG-BS results.

### 2.5 ANI-2X/CG-BS Calculations

ANI is a machine learning model developed by the Roitberg group at the University of Florida that uses an Atomic Environment Vector to build a neural network.^18^ ANI applies a modified version of Behler and Parrinello symmetry functions to construct atom-centered environment vectors for each atom which reflects the varying contributions to conformational energy by different chemical environments surrounding each atom.^18^ The ANI-1 potential was trained on 22 million molecular conformations randomly selected from 57,000 different small molecules using energies calculated with the ωB97X DFT method and 6-31G(d) basis set in Gaussian 16. Additionally, a bound-learning-based ANI potential (ANI-1x) built on only 5 million conformations outperformed the original ANI-1. ANI-1/1x can only be applied to molecules containing C, H, N, and O elements. The latest ANI-2X expanded its applicability to seven chemical elements (C, H, N, O, S, F, and Cl) by training on 8.9 million molecular conformations with bound learning torsion refinement.^19^

The geometry optimization step is performed using an internal program to conduct conjugated gradient optimization with backtracking line search.^9^ The CG-BS algorithm effectively overcomes the computational bottleneck of traditional optimization algorithms in optimizing the rough PES. This algorithm uses the energy and force provided by the ANI to update the coordinates of the molecules and iterates according to Wolfe line search conditions to prevent too many searching steps until the convergence condition is reached. The optimization direction is determined by calculating the gradient direction and using the line search method to select the optimal step size. In this way, ANI works with the optimization algorithm to produce more accurate and reliable molecular geometries, converging to better results within fewer iteratives. We conducted ANI-2X/CG-BS optimization applying the same default convergence criteria as in Gaussian 16 optimization; i.e., the maximum and mean displacements are 0.0018 and 0.0012 atomic units, and the maximum and mean RMS forces are 0.00045 and 0.00030 atomic units, respectively. A very large value for the “maximum iterative steps” parameter, 10,000, was utilized so that all geometry optimizations can be converged successfully.

## 3 Results and Discussion

### 3.1 Evaluation of ANI-2x/CG-BS with a 231-Molecule Data Set

**Figure 2:**
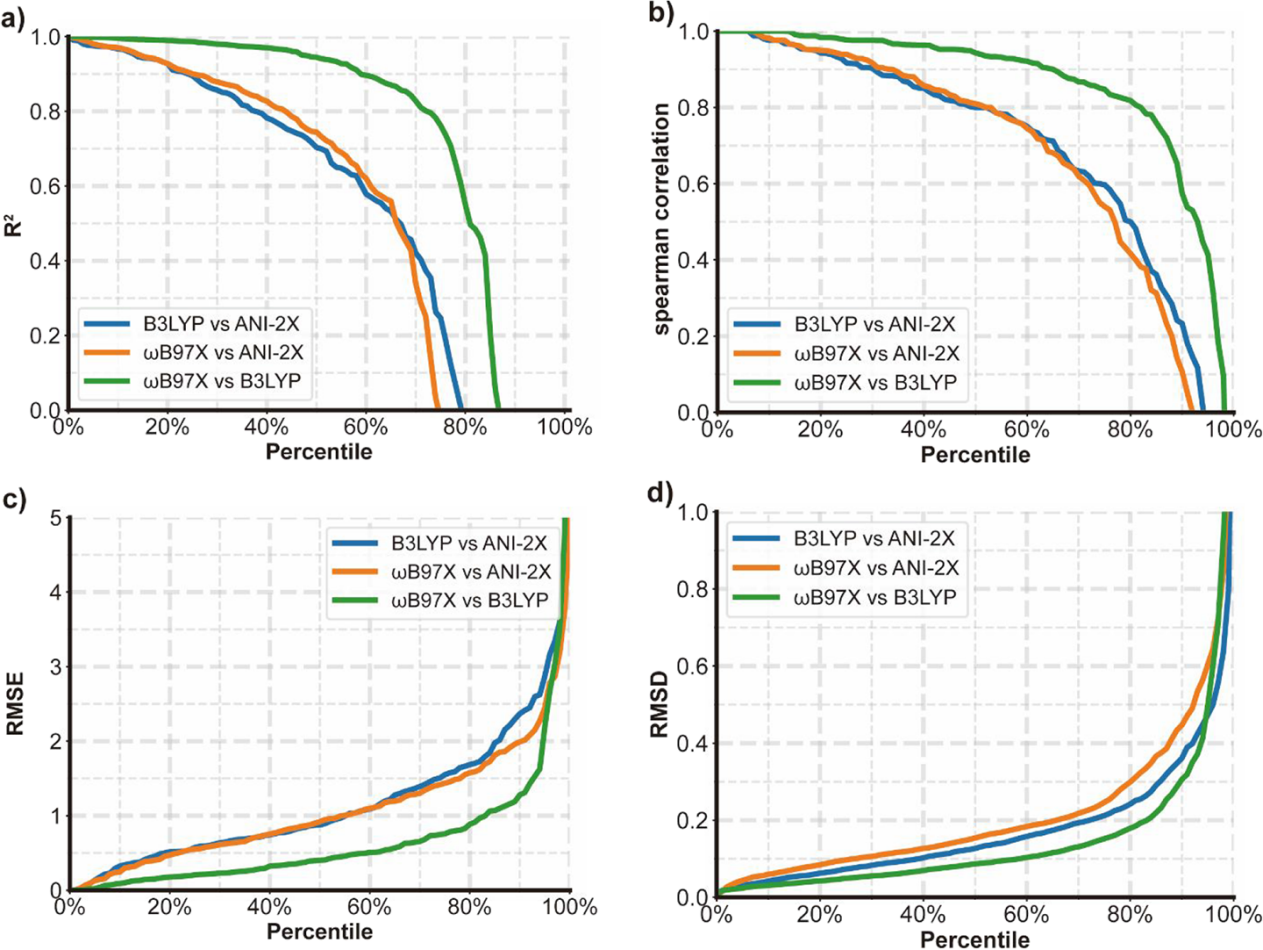
Comparison of the conformational energies between ANI-2X/CG-BS and the ab initio models (ωB97X and B3LYP) after geometry optimization. Higher *R*^2^ and Spearman values indicate superior fitting results, while lower RMSE and RMSD values indicate smaller deviations. Spearman and *R*^2^ values are truncated at 0 for low values, while RMSE and RMSD values range from 0 to 5 kcal/mol and 1 Å, respectively.

231-molecule Benchmark Data set 1 includes molecules consisting of the basic C, H, O, and N atoms as well as the extended S, F, and Cl atoms. The ANI-2x method, as an extension of the ANI-1x approach, offers extra support for the last three elements. We chose Benchmark Data set 1 to objectively evaluate the performance of ANI-2x/CG-BS. A comparative analysis was performed to assess the relative conformation energies of ab initio models ωB97X and B3LYP, as well as ANI-2X/CG-BS. The assessment applied energy metrics such as RMSE, correlation metrics including *R*^2^ and ρ, and the evaluation of conformational deviation before and after the ANI-2X/CG-BS optimization as measured by RMSD (Figure 2). The statistical outcomes of those performance metrics, as well as computational processing time on the central processing unit (CPU), are summarized in Table 1.

**Table 1:** Statistics for Conformation Energy, Correlation, Structure Deviation, and CPU Calculation Time for 1882 Conformations in 231 Ligand Molecules^a^

metrics		mean	median	standard deviation
R^2^	ωB97X vs B3LYP	0.89	0.97	0.17
	ωB97X vs ANI-2X	0.77	0.84	0.22
	B3LYP vs ANI-2X	0.72	0.79	0.24
ρ	ωB97X vs B3LYP	0.85	0.94	0.26
	ωB97X vs ANI-2X	0.65	0.81	0.42
	B3LYP vs ANI-2X	0.68	0.80	0.36
RMSE (kcal/mol)	ωB97X vs B3LYP	0.66	0.40	0.95
	ωB97X vs ANI-2X	1.07	0.92	0.79
	B3LYP vs ANI-2X	1.16	0.88	0.93
RMSD (Å)	ωB97X vs B3LYP	0.15	0.09	0.22
	ωB97X vs ANI-2X	0.22	0.15	0.22
	B3LYP vs ANI-2X	0.17	0.13	0.16
CPU time per conformation (s)	ANI-2X	28.38	23.99	18.35
	ωB97X	20,305	14,912	20,100

a Molecules with negative *R*^2^ values were omitted, leaving ωB97X versus B3LYP with 199 ligands, ωB97X versus ANI-2X with 173 ligands, and B3LYP versus ANI-2X with 183 ligands. The statistics for RMSE and RMSD were also generated just for molecules with non-negative *R*^2^ values.

The optimization results of ANI-2X/CG-BS, as illustrated in Figure 2a,b, exhibit performance comparable to that of ωB97X and B3LYP. The mean *R*^2^ value of ANI-2X/CG-BS compared with that of ωB97X is observed to be 0.84, while the ρ value is approximately 0.81. As shown in Figure 2c, the ANI-2X-optimized structures exhibit a robust and accurate energy result compared with the ωB97X result, which has a deviation below 2 kcal/mol for about 90% of the molecules, whereas the deviation of ANI-2X from the B3LYP result reaches about 2.4 kcal/mol at this 90% point. The RMSD results suggest that ANI-2X-optimized structures show a higher degree of similarity to the B3LYP-optimized structures, which serves as the initial configuration for geometric optimization in comparison to the ωB97X-optimized results (Figure 2d). The ANI-2X calculations were performed with a total wall time of 14.85 h, resulting in a mean value for the time cost of around 28 s. This represents a significant reduction in time cost, amounting to approximately 1/700th of the time cost spent by the ωB97X optimizations, as illustrated in Table 1.

### 3.2 Evaluation of ANI-2x/CG-BS with a 8992-Molecule Data Set

We evaluated ANI-2x/CG-BS using the much larger Benchmark Data set 2 for which the B3LYP optimization was performed for 71,343 total conformations of 8992 ligand molecules (Table 2). The SMILES strings for Benchmark Data set 1 molecules are provided in Supplementary Table 1.

**Table 2:** Statistics for Conformation Energy, Correlation, Structure Deviation, and CPU Calculation Time for 71,343 Conformations in 8992 Ligand Molecules^a^

metrics	mean	median	standard deviation
*R*^2^	0.74	0.82	0.24
ρ	0.78	0.85	0.21
RMSE (kcal/mol)	1.12	0.94	0.95
RMSD (Å)	0.17	0.13	0.16
CPU time per conformation (s)	29.89	25.34	19.58

a Molecules with negative *R*^2^ values were omitted, leaving B3LYP vs ANI-2X with 7070 ligands. The statistics for RMSE and RMSD were also generated just for molecules with non-negative *R*^2^ values.

**Figure 3:**
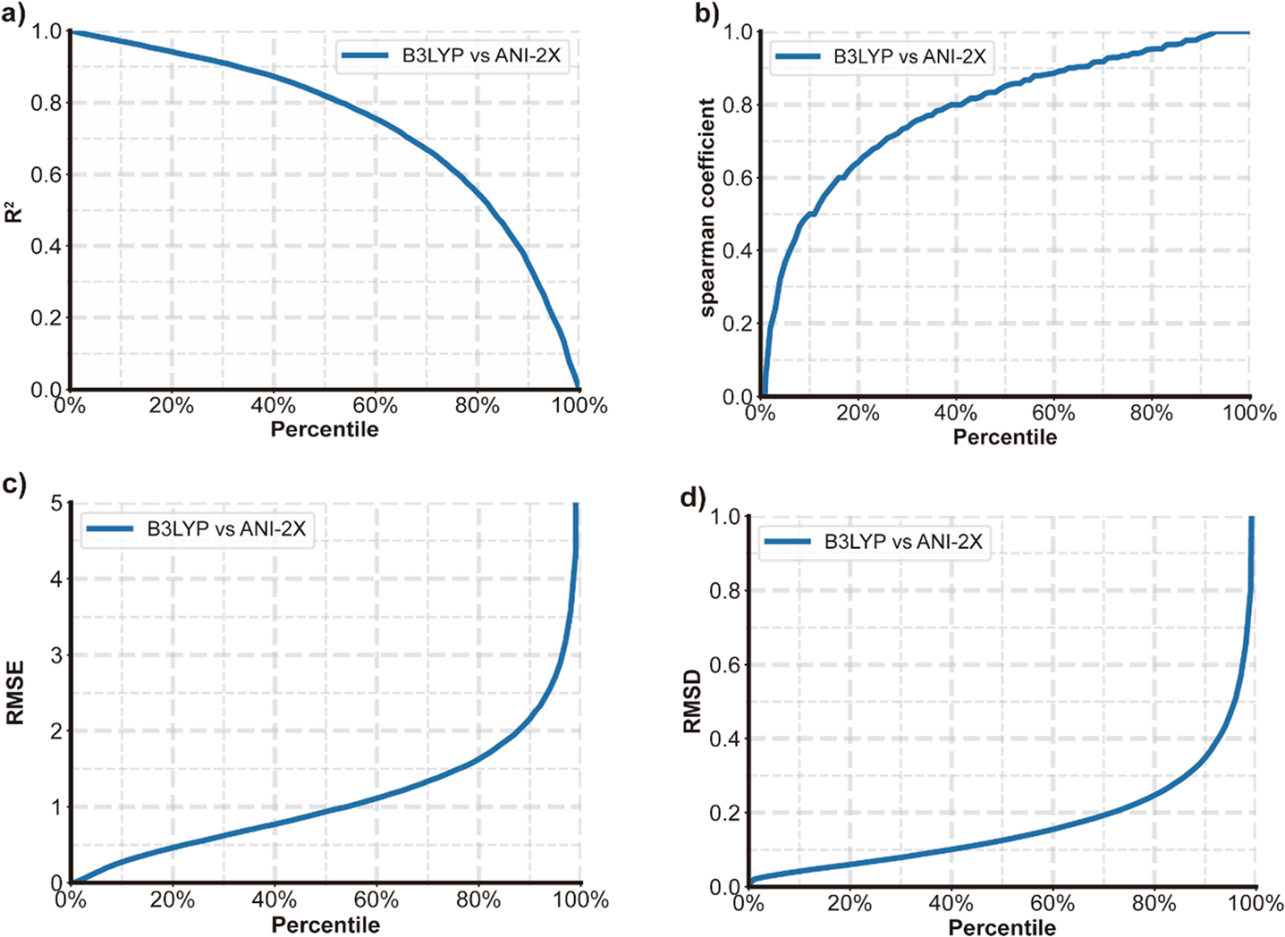
*R*^2^, RMSE, ρ, and conformational RMSD between ANI-2X and B3LYP results.

The general computational performance of ANI-2X/CG-BS was also assessed using various performance metrics, as depicted in Figure 3. The mean *R*^2^ and ρ values, 0.74 and 0.78, respectively, indicate that ANI-2X/CG-BS achieves a satisfactory performance in reproducing the B3LYP energies, albeit the targeted ab initio model in ANI-2X development is ωB97X. Upon comparing the statistical data between the two molecular sets, it becomes evident that ANI-2X exhibits a similar performance to B3LYP: the median RMSE values are 0.88 and 0.94 kcal/mol for Benchmark Data set 1 and Benchmark Data set 2, respectively; the median CPU timings are 23.99 and 25.34 s for the two corresponding data sets; and the RMSD for measuring the geometry deviations is the same, 0.13 Å. The slightly better values of the performance metrics for Benchmark Data Set 1 are statistically insignificant. Overall, ANI-2X/CG-BS has demonstrated satisfactory and reliable computational performance for both Benchmark Data set 1 and Benchmark Data set 2, regardless of their sample sizes.

### 3.3 Schemes of Filtering and Clustering Protein Data Bank X-Ray Ligands

**Figure 4:**
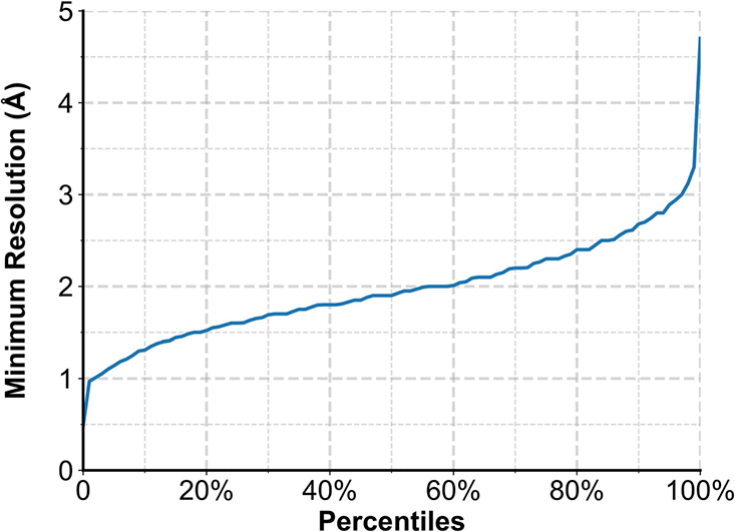
Percentile distribution of resolutions for 36,104 PDB ligand-bound X-ray structures.

Since a PDB ligand may correspond to multiple PDB structures, only the one with the minimum resolution is selected. Figure 4 shows the distribution of the resolutions of those PDB X-ray structures, and 97% of them are considered to have medium to high quality with resolutions below 3 Å.^20^ The mean value of the resolutions is 1.96 Å, with a standard deviation of 0.52 Å.

**Figure 5:**
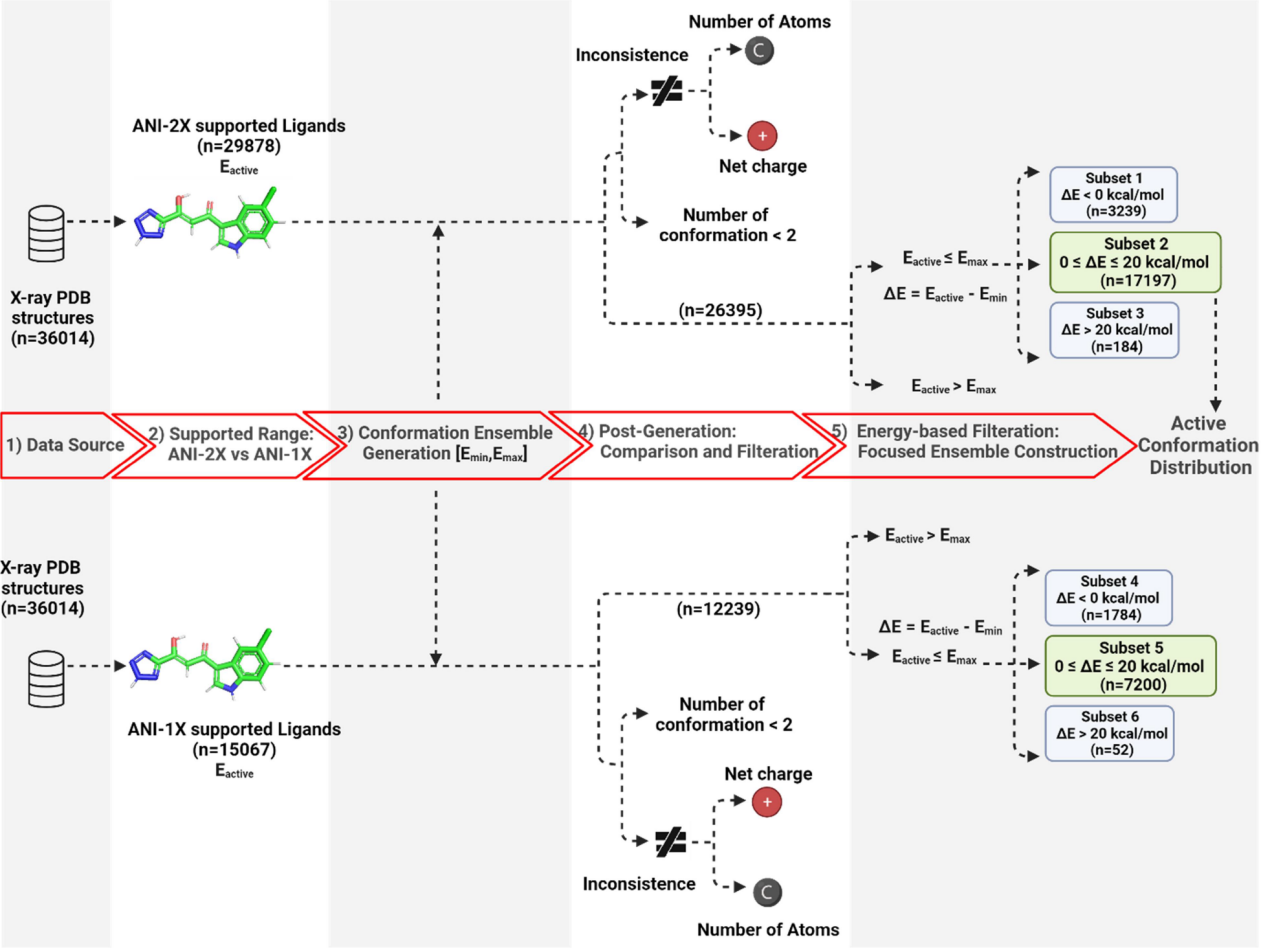
Filtering and clustering schemes for the 36,014 crystal ligands in the PDB.

As shown in Figure 5, out of the total 36,014 entries, 15,067 consist of elements (C, H, O, and N) all supported by ANI-1X, while 29,878 ligands consist of elements (C, H, O, N, S, F, and Cl) all supported by ANI-2X. These entries were subsequently subjected to conformation generation using Omega2 software. We then calculated the energy for each ligand’s conformation ensemble. The global minimum energy *E*_global_(*E*_min_) and maximum conformation energy *E*_max_ were applied for energy comparison. By default, Omega2 automatically fix “problematic” molecules, such as filling in open valences and changing protonate states. Moreover, the atom names, atom types, bond types, as well as atom sequence order may be changed for the generated conformations. We used canonical SMILES^21^ and fingerprints including ECFP,^22^ FP4, and MACCS^23^ to check whether the bound conformation and the Omega2-generated conformations belong to the same molecule. However, we found that none of those methods can guarantee success for all of the molecules. We applied match_atomname, a program from the Antechamber package to reliably filter out those molecules that the bound conformation and the Omega2-generated conformations have different atomic paths. Of note, match_atomname enumerates all possible element-based atomic paths for a given molecule. By applying the match_atomname filter, a total of 26,395 entries survived.

Next, we cluster the 26,395 ligands into three subsets: in Subset 1, 3239 ligands have bound conformation energy smaller than the global conformation of the Omega2-generated conformational ensemble; in Subset 2, 17,197 ligands have their bound conformation energies larger than global conformation energies, but their energy differences, Δ*E* = *E*_active_ – *E*_global_, are within 20 kcal/mol; in Subset 3, 5775 ligands have Δ*E* larger than 20 kcal/mol. We focused on Subset 2 which is the predominant scenario to analyze the distribution of bound conformations in conformational ensembles. We neglected Subset 3 molecules to eliminate the statistical instability. The ligand IDs and PDB entry IDs of Subsets 1–3 are listed in Table S2. The same filtering procedures were also applied for the ANI-1X-supported entries. As shown in Figure 5, the ANI-2X method has doubled the range of supported entries in the whole filtering procedure.

**Figure 6:**
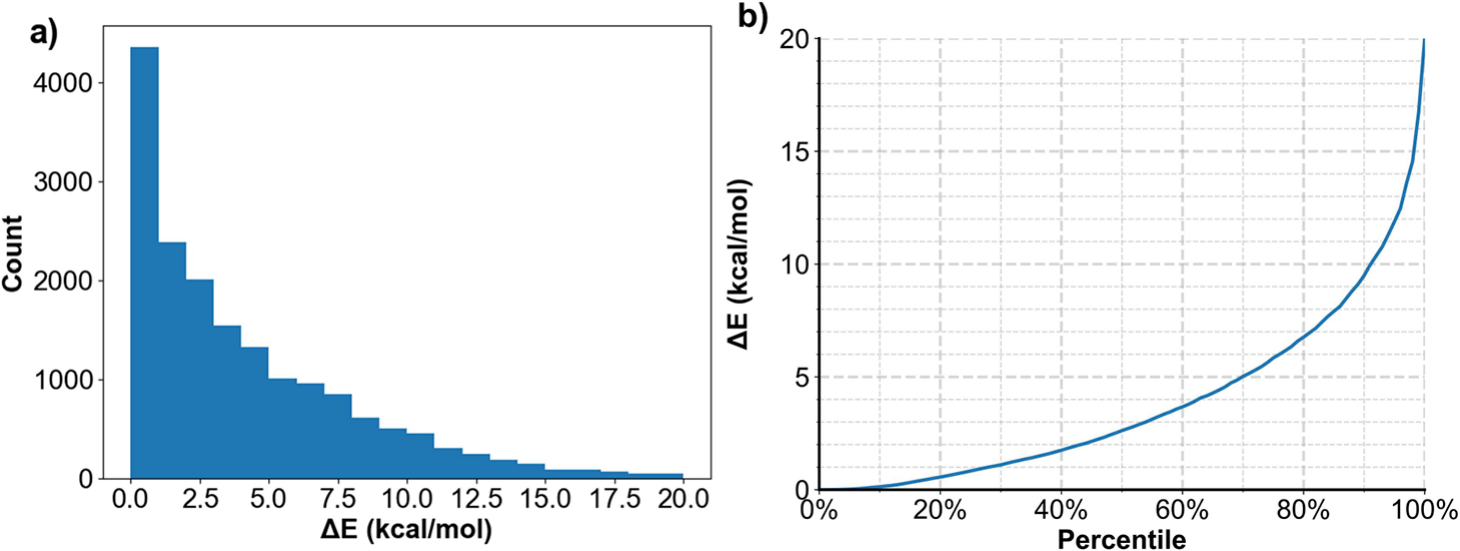
Conformational energy distributions for crystal ligands. The energy difference Δ*E* = *E*_active_*– E*_global_, where *E*_active_ is the ANI-2X/CG-BS energy for the bound conformation, *E*_global_ is the ANI-2X/CG-BS energy of the global conformation in the Omega2-generated conformational ensemble. (a) Distribution of the counts of PDB ligands with Δ*E* between 0 and 20 kcal/mol; (b) accumulated percentiles with Δ*E* increasing from 0 to 20 kcal/mol.

For Subset 2, 50% of the molecules have generated a maximum of 200 conformations, whereas 95% of the molecules have generated at least 5 conformations. The distribution of the bound ligands among the conformational ensembles based on their ANI-2X/CG-BS energies is shown in Figure 6. The Δ*E* values are 0.98, 2.91, and 6.29 kcal/mol for the first quartile, median, and the third quartile, respectively (Figure 6a). For 90% of the ligands, their relative energies Δ*E* are within 9.85 kcal/mol, as shown in Figure 6b.

ANI-2X/CG-BS optimization can lead to a starting conformation into a local/global minimum in the PES. We calculated the RMSD values before and after geometry optimizations for all 2.25 million conformations. The average RMSD is 0.600 Å, with a standard deviation of 0.348 Å. As far as the bound conformations are concerned, the average RMSD value is 0.534 ± 0.353 Å (Table 3).

**Table 3:** Statistics of CPU Time and RMSD Value before and after ANI-2X/CG-BS Optimizations

metric	conformation set	mean	median	standard deviation
RMSD (Å)	2.25 million	0.600	0.536	0.348
RMSD (Å)	17,197 bound conformations	0.534	0.451	0.353
CPU time per conformation (s)	2.25 million	99.33	87.89	63.18

For all the 2.25 million conformations of the 17,193 ligands, the total CPU time for ANI-2x/CG-BS calculations is 62,061 h with Intel Xeon Gold 6126 CPUs, i.e., 99.33 s per conformation on average.

### 3.4 ANI-2X/CG-BS Is a Promising Tool for Conformational Library Design

As shown in Figure 5, the majority of PDB ligands belong to Subset 2. For the 17,197 Subset 2 conformational ensembles, 53% of conformations have their relative conformation energies larger than 20 kcal/mol. Thus, removal of those high-energy conformations can result in great time and effort savings for virtual screening or docking simulation. We can also apply different thresholds to eliminate the high-energy conformations. For example, if a threshold of 10 kcal/mol is applied, 57% conformations can be eliminated but with a cost of 10% bound conformations also being discarded. If a threshold of 6 kcal/mol is applied, 64% of high-energy conformations along with 26.6% of bound conformations are eliminated. If a threshold of 2.91 kcal/mol is applied, the median of Δ*E* of the bound conformations, 50% bound conformations are eliminated with a gain of removing 73% high-energy conformations from the conformational ensembles.

To obtain a refined look of the threshold for subsets of molecules, we listed the median Δ*E* as well as Δ*E* at the first and third quartiles and 90% for subsets with varied molecular sizes (Table 4).

**Table 4:** Statistics on the Relative Energies (kcal/mol) of the Bound Conformations for 17,197 PDB Ligands in Subset 2^a^

atom count	count	1st quartile	median	3rd quartile	90%	mean
<30	2399	0.18	1.32	3.37	6.48	2.32 ± 2.78
30–40	3281	0.48	1.98	4.58	8.03	3.11 ± 3.38
40–50	4039	0.91	2.56	5.29	8.36	3.60 ± 3.49
50–60	3382	1.47	3.47	6.50	9.75	4.44 ± 3.79
60–70	1990	1.95	4.26	7.78	10.98	5.23 ± 4.11
>70	2106	3.39	6.59	10.39	13.76	7.20 ± 4.69

a Each ligand belongs to 1 of 6 groups according to its atom count.

**Figure 7:**
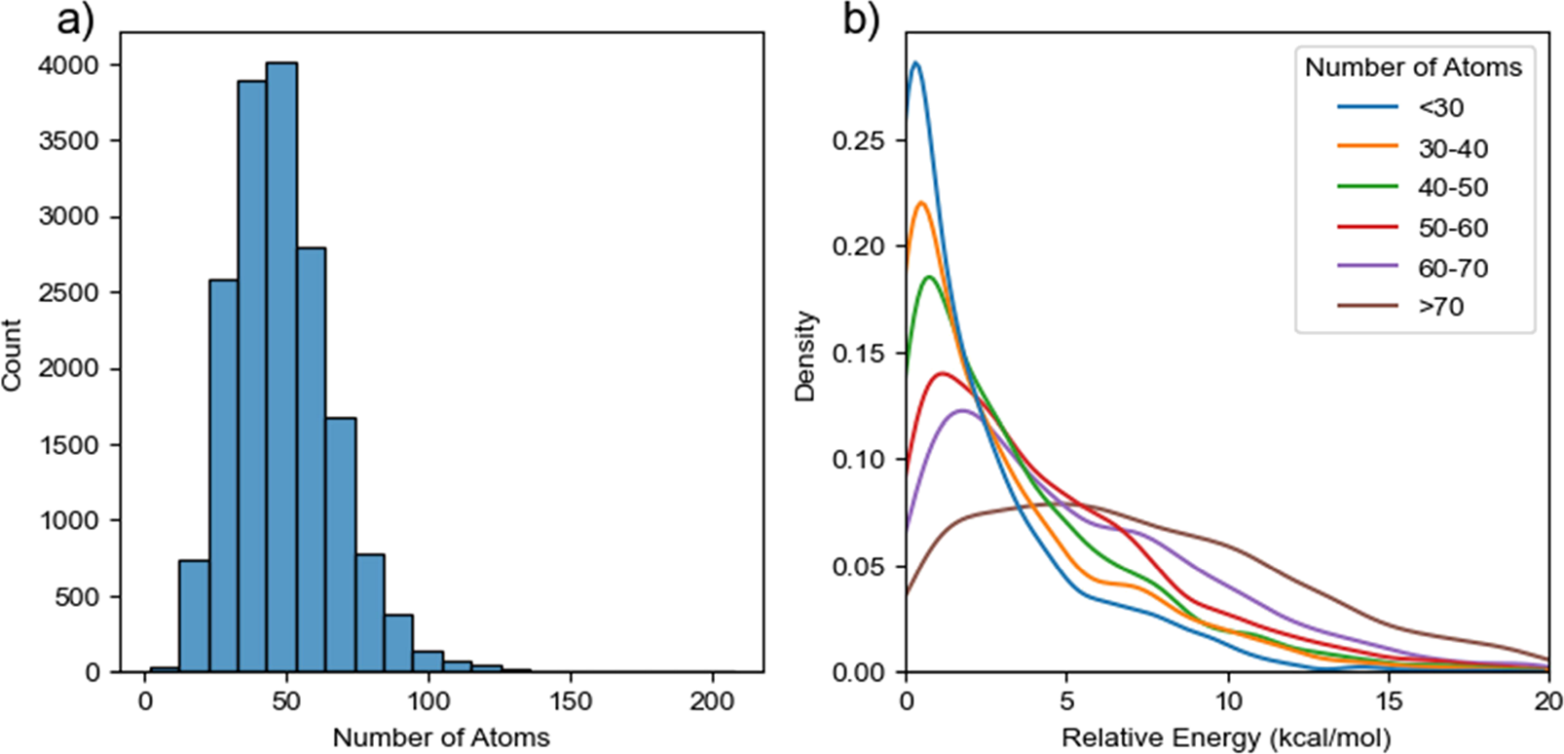
(a) Atom count distribution for 17,197 PDB ligands in Subset 2; (b) relative conformational energy distribution for PDB ligands with different sizes measured by the atom count.

The 90% threshold was 6.48 kcal/mol for ligands with atom counts less than 30, while this threshold increased to 13.76 kcal/mol for ligands with atom counts more than 70. The distribution of atom counts for PDB ligands and the distributions of the relative energies for six ligand subsets are illustrated in Figure 7. Apparently, the majority of PDB ligands have numbers of atoms around 50 with a standard deviation of 18.6 and a mean value of 48.6 (Figure 7a). It is shown that a more and more flattened distribution of relative energies for the bound conformations is observed when the molecular size gradually increases (Figure 7b). Thus, the molecular size measured by the number of atoms greatly influences the bound conformational energy distribution, and the skewed distributions might reflect the fact that those molecules, even in the same subset, have different degrees of structural flexibility and complexity.

**Figure 8:**
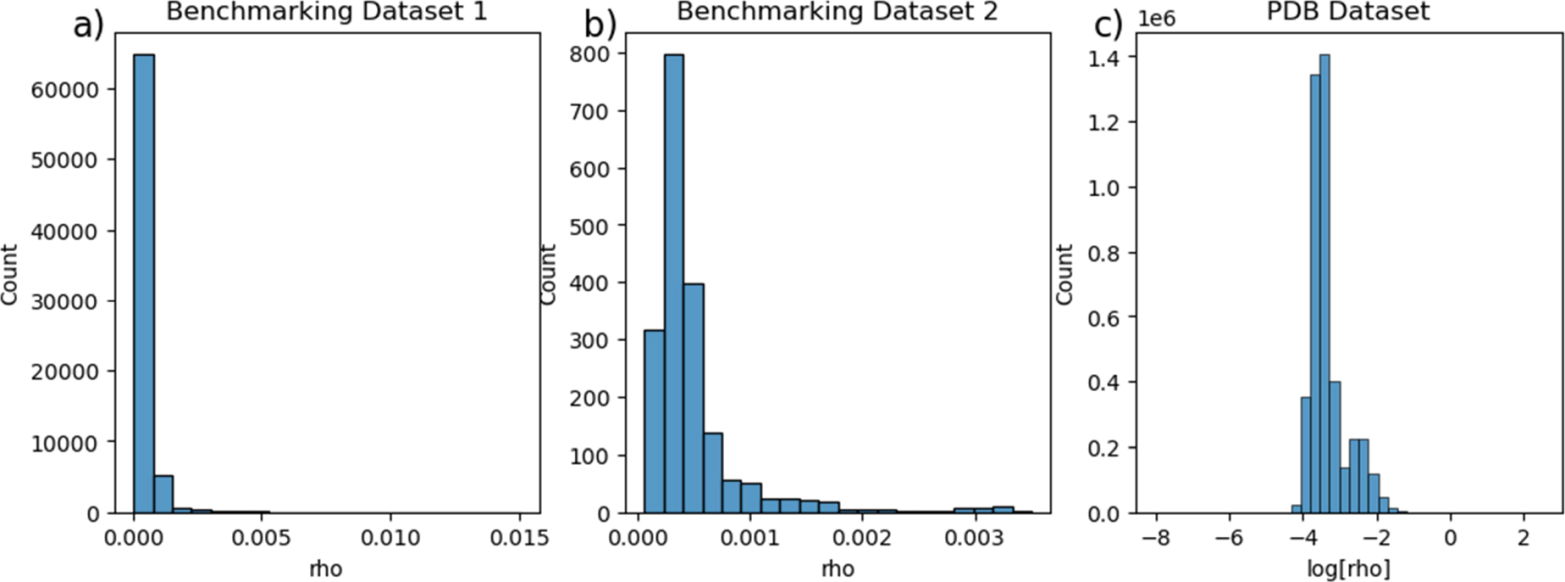
Rho values distribution for three data sets. (a) Benchmarking Data set 1; (b) Benchmarking Data set 2; (c) PDB ligand data set.

The accuracy of ANI potential can be further evaluated by the rho parameter, which is the standard deviation of ANI potentials that are predicted by an ensemble of models.^9^ This value is weighted by the square root of the number of atoms present in each molecule. When the value of rho is less than 0.23 kcal/mol, almost 98% of the molecules within their chemical set exhibit predicted errors fewer than 1.5 kcal/mol. The rho value distributions for Benchmarking Data set 1, Benchmarking Data set 2, and PDB Data set are shown in Figure 8. In Figure 8c, for a comprehensive demonstration purpose, we added 10 × 10^–20^ to the rho value to remove zero values and turned it into a log scale. Most of the rho values are within 10 × 10^–4^ to 10 × 10^–2^ kcal/mol, which demonstrates the high reliability of ANI prediction.

### 3.5 The ANI-2X/CG-BS Performance Is Net Charge-Dependent

Benchmark Data set 1 comprises a total of 1882 conformations for 231 molecules, including 211 neutral and 20 charged molecules. The performance statistics for the 1709 conformations of neutral molecules and 173 conformations of the charged molecules are listed in Table 5. It is evident that the ANI-2X/CG-BS performance of neutral molecules surpasses that of charged molecules according to various performance metrics. When comparing ANI-2X to ωB97X and ANI-2X to B3LYP, the RMSE values are lower for both the neutral and charged molecules in the former.

**Table 5:** Statistics of the ANI-2X/CG-BS Performance for the Charged and Neutral Molecules in Benchmark Data Set 1

metrics	charge type		mean	median	standard deviation
*R*^2^	meutral	ωB97X vs B3LYP	0.90	0.97	0.16
		ωB97X vs ANI-2X	0.78	0.85	0.21
		B3LYP vs ANI-2X	0.74	0.81	0.23
	charged	ωB97X vs B3LYP	0.65	0.61	0.27
		ωB97X vs ANI-2X	0.58	0.59	0.23
		B3LYP vs ANI-2X	0.60	0.63	0.19
ρ	neutral	ωB97X vs B3LYP	0.86	0.95	0.26
		ωB97X vs ANI-2X	0.66	0.82	0.41
		B3LYP vs ANI-2X	0.65	0.81	0.42
	charged	ωB97X vs B3LYP	0.73	0.84	0.26
		ωB97X vs ANI-2X	0.50	0.61	0.50
		B3LYP vs ANI-2X	0.56	0.69	0.46
RMSE (kcal/mol)		ωB97X vs B3LYP	0.53	0.37	0.75
	neutral	ωB97X vs ANI-2X	1.03	0.88	0.77
		B3LYP vs ANI-2X	1.07	0.86	0.80
	charged	ωB97X vs B3LYP	2.02	1.42	1.59
		ωB97X vs ANI-2X	1.56	1.65	0.86
		B3LYP vs ANI-2X	2.09	1.86	1.56
RMSD (Å)		ωB97X vs B3LYP	0.14	0.08	0.21
	neutral	ωB97X vs ANI-2X	0.21	0.15	0.22
		B3LYP vs ANI-2X	0.17	0.12	0.16
	charged	ωB97X vs B3LYP	0.22	0.13	0.24
		ωB97X vs ANI-2X	0.27	0.18	0.22
		B3LYP vs ANI-2X	0.24	0.18	0.15
CPU time per conformation (s)	neutral	ANI-2X	27.59	23.51	17.63
		ωB97X	18899	14420	17141
	charged	ANI-2X	36.21	31.67	22.77
		ωB97X	34203	23935	35877

Benchmark Data set 2 has 8155 neutral ligands and 837 charged molecules. Table 6 lists the performance of ANI-2X/CG-BS for both the neutral and charged molecules. It is reasonable to observe that ANI-2X/CG-BS in comparison with B3LYP achieves better performance for the neutral molecules than the charged molecules. The mean RMSE value for the charged molecules, 2.20 kcal/mol, is doubled compared to the mean RMSE value of the neutral molecules. The larger RMSE values for the charged molecules are expected given the fact that the ANI model was trained using neutral molecules. As far as the changes of geometries after ANI-2X/CG-BS optimizations are concerned, the RMSD values exhibit a 50% increase from 0.17 Å for the neutral molecules to 0.24 Å for charged molecules. In brief, we concluded that ANI-2X/CG-BS exhibited a higher level of suitability for performing geometry optimization for neutral molecules. It is expected that it also performs well for the charged molecules with a future machine learning potential that takes the charged molecules into consideration during the training process.

**Table 6:** Statistics on Charged and Neutral Molecules in Benchmark Data Set 2

metrics	charge type	mean	median	standard deviation
*R*^2^	neutral	0.59	0.74	0.37
	charged	0.48	0.57	0.36
ρ	neutral	0.64	0.80	0.45
	charged	0.55	0.71	0.48
RMSE (kcal/mol)	neutral	1.06	0.86	1.48
	charged	2.20	2.02	2.03
RMSD (Å)	neutral	0.17	0.12	0.17
	charged	0.24	0.19	0.20
CPU time per conformation (s)	neutral	29.18	24.65	19.21
	charged	37.04	32.60	21.76

## 4 Conclusions

The bound conformations of ligand–receptor binding complexes archived in PDB play a vital role in drug-target interactions. Flexible ligand docking is a widely used approach to explore the bound conformation of a ligand when it binds to a receptor. This process requires computational algorithms to efficiently filter out conformations that are less likely to be the bound conformation. Moreover, in conformational library design, eliminating those high-energy conformations which are less likely be the bound conformations can save not only disk space but also computational resource for virtual screenings. However, to eliminate the high-energy conformations, a reliable potential is needed. Herein, we use ANI-2X machine learning potential in combination with an internal conjugate gradient with a backtracking line search algorithm (ANI-2X/CG-BS) to accurately calculate the potential energies of the conformation ensembles. The performance of ANI-2X/CG-BS was critically evaluated using two molecular sets, Benchmark Data set 1 and Benchmark Data set 2. ANI-2X/CG-BS demonstrated comparable accuracy to two DFT models but with a much short computer time by reducing a prior QM calculation process. The mean RMSE values are about 1 and 1.5 kcal/mol for the general performance of the neutral and charged molecules, respectively. Given that ANI-2X’s training was on neutral molecules, the observed deviations for charged molecules are expected. The speedup of the computer time is about 700–1000 folds. Encouraged by the excellent performance of ANI-2X/CG-BS, we collected 29,878 bound conformations for the same number of PDB ligands and generated conformational ensembles using Omega2 software. After a series of necessary filtering processes, 17,197 ligands which have in total 2.25 million conformations survived. The relative potential energies of bound conformations to the global conformations of the conformational ensembles were calculated and analyzed. The median relative potential energy is 2.91 kcal/mol, and 90% of the molecules have relative energy values smaller than 9.85 kcal/mol. By applying a threshold of 10 kcal/mol for eliminating high-energy conformations, we can remove 58% conformations from the conformational ensembles. We are also aware of certain limitations in the ANI-2X/CG-BS method, which is mainly associated with ANI-2X. For example, ANI-2X has inferior performance when dealing with charged molecules as ANI-2X was trained only using neutral molecules, albeit CG-BS minimization can reduce the prediction errors for the charged molecules. Additionally, molecules containing elements not covered by ANI-2X cannot be studied by using this method. Also, recent advancements in the field emphasize the importance of efficient conformer selection methodologies. For instance, the Autonomous Graph Based Clustering (AG) tool, developed by Tanemura et al., offers a promising approach for conformer clustering followed by energy calculations using machine learning techniques.^24^ Such methods can potentially streamline the process by reducing the number of conformers that need detailed energy calculations, allowing for a more comprehensive exploration of the conformational space while optimizing computational resources. It is an appealing idea to apply this method to treat the Omega2-generated conformational ensemble prior to ANI-2x/CG-BS calculation. Despite these limitations, ANI-2X/CG-BS is a promising tool for conformational library design; the information on the distribution of bound conformations in conformational ensembles can guide us to eliminate ligand conformations that are unlikely to form a bound conformation. This information is very useful in conformational library design and docking algorithm development.

## Data Availability

All the energy data collected was calculated by ANI-2X in combination with CG-BS algorithms. The data analysis was conducted with Python3. The ANI-2X/CG-BS code can be downloaded freely from https://github.com/junmwang/pyani_mmff. The ANI-2X test set and PDB ligand molecules for calculation can be downloaded from 10.5281/zenodo.8415657.

## Supporting Information Available

Smiles data for Benchmark Data set 2 (XLSX)
Ligand IDs and the corresponding PDB entry IDs to obtain the bound conformations (XLSX)

## Abbreviation

ANI: Accurate Neural network engIne for molecular energies
CG-BS: Conjugate Gradient with Backtracking Line Search
DFT: density functional theory
MM: molecular mechanics
R^2^: *R*-square
RMSD: root mean square deviation
RMSE: root mean square error
ρ: Spearman’s rank correlation coefficient
PDB: Protein Data Bank
